# A novel procedure for the quantitative analysis of metabolites, storage products and transcripts of laser microdissected seed tissues of *Brassica napus*

**DOI:** 10.1186/1746-4811-7-19

**Published:** 2011-06-30

**Authors:** Silke Schiebold, Henning Tschiersch, Ljudmilla Borisjuk, Nicolas Heinzel, Ruslana Radchuk, Hardy Rolletschek

**Affiliations:** 1 Leibniz Institute of Plant Genetics and Crop Plant Research (IPK), Corrensstr. 3, 06466 Gatersleben, Germany

## Abstract

**Background:**

The biology of the seed is complicated by the extensive non-homogeneity (spatial gradients) in gene expression, metabolic conversions and storage product accumulation. The detailed understanding of the mechanisms underlying seed growth and storage therefore requires the development of means to obtain tissue-specific analyses. This approach also represents an important priority in the context of seed biotechnology.

**Results:**

We provide a guideline and detailed procedures towards the quantitative analysis of laser micro-dissected (LM) tissues in oilseed rape (*Brassica napus*). This includes protocols for laser microdissection of the seed, and the subsequent extraction and quantitative analysis of lipids, starch and metabolites (sugars, sugar phosphates, nucleotides, amino acids, intermediates of glycolysis and citric acid cycle). We have also developed a protocol allowing the parallel analysis of the transcriptome using *Brassica*-specific microarrays. Some data are presented regarding the compartmentation of metabolites within the oilseed rape embryo.

**Conclusion:**

The described methodology allows for the rapid, combined analysis of metabolic intermediates, major storage products and transcripts in a tissue-specific manner. The protocols are robust for oilseed rape, and should be readily adjustable for other crop species. The suite of methods applied to LM tissues represents an important step in the context of both the systems biology and the biotechnology of oilseeds.

## Background

Both the human and animal diet depends heavily, either directly or indirectly, on plant seeds, which are also used as a raw material for a number of industrial applications. A substantial research effort has therefore been focused on gaining a fuller understanding of seed development, and specifically of the mechanisms underlying the accumulation of the storage products oil, protein and starch. The seed is a complex structure, in which the various constituent organs (seed coat, endosperm and embryo) each have their specific function, determined by the integrated activity and specialization of groups of tissues/cells.

Treating the seed as a homogeneous entity inevitably ignores variation in the localized distribution of metabolites, gene expression, etc., and thus is not appropriate for investigating the spatial regulation of metabolism. Laser-assisted micro-dissection (LM), introduced by Emmert-Buck et al. [1], was developed as a means of sampling small groups or even single cells within plant tissues [2-4]. In this technique, the target is microscopically identified within a thin section, and then isolated via a computer-guided UV laser.

Currently, LM applications in plant research are predominantly concerned with the analysis of localized transcript abundance [4-9], including within the *Arabidopsis thaliana *embryo [10-12] or endosperm [13,14], and the barley caryopsis [15,16]. LM has also been successfully applied to analyse localized enzyme activity [17], identify proteins [18,19] and metabolites [17,20-23].

The combination of transcript profiling and biochemical analysis is particularly challenging, as these require contrasting fixation/extraction procedures [24]. However, such approaches would allow relating gene expression to the actual levels of metabolites and/or storage products in particular regions of the seed, making the link from genes to storage activities. Apart from the study of Thiel et al. [25] combining HPLC-based analysis of free amino acids with analysis of transcripts for the nucellar projection/endosperm transfer cell complex of barley caryopses, there are no studies published providing versatile analytical tools targeting seed tissues.

Oilseed rape (*Brassica napus*) is one of the leading temperate crops grown as a source of oil, and although much of the global physiology occurring over the course of its seed development is well understood [26-31], little attention has been paid to studying metabolism within specific tissues of the developing seed. The aim of the present research was to develop a set of procedures suitable for the parallel assessment and quantitation of metabolites, storage products and transcripts in LM tissues from developing seeds. We have established the necessary tools for achieving an integrated quantitative analysis of LM tissues and have demonstrated the application of LM for the study of tissue-specific metabolism in the oilseed rape seed.

## Results and Discussion

### Preparation and collection of tissue-specific samples via laser microdissection from oilseed rape seed

**Figure 1:**
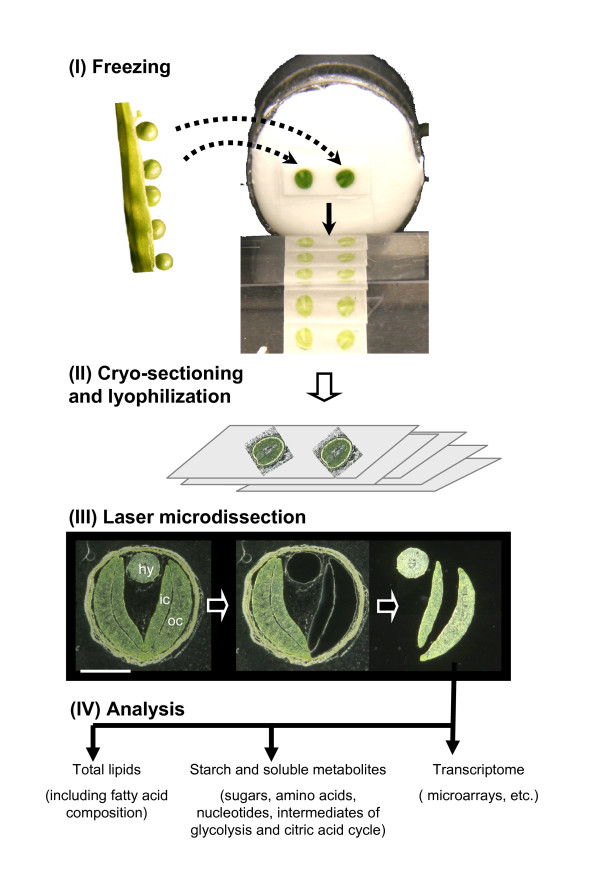
**Experimental workflow**. Schematic overview of the laser-microdissection based analysis of metabolites, storage products and transcripts in seeds of *Brassica napus*: (**I**) Seeds are removed out of the silique and immediately snap-frozen in liquid nitrogen. Two seeds mounted for cryosectioning are cut in 20 μm thick cross sections. (**II**) Sections are transferred to PET membrane slides and freeze-dried for one week. (**III**) Tissues of interest are laser microdissected from the cross sections. Light microscopy pictures show a cryosection of a 28 day old rape seed (bar = 1 mm) (**IV**) Isolated tissues are processed in parallel for subsequent biochemical and transcript analyses. Abbreviations: hy-hypocotyl; ic-inner cotyledon; oc-outer cotyledon.

The first requirement was to optimize the preparation of tissue from the oilseed rape seed, appropriate for both transcriptome and biochemical analysis. Standard fixation and embedding procedures have been designed to preserve the morphological integrity of the sample, as well as to guarantee a sufficient quality of the RNA needed for gene expression analyses. However, these procedures entail the loss of many metabolites from the sample [20,25]. A cryogenic approach was therefore adopted in which the whole seed was first snap-frozen in liquid nitrogen, before sectioning without any additional chemical treatment. Sections of thickness 15, 20 or 30 μm were mounted either on standard microscope glass slides, or on various types of membrane-covered slides. The sections were dried at -20°C and stored at -80°C until required. The resulting morphology was adequate to distinguish between the various seed organs and even, to some extent at least, to enable the recognition of cell types such as the vascular tissue inside the hypocotyl, or the various cell layers of the seed coat. The dried sections were then subjected to LM (Figure 1), in which the target was first identified microscopically and then excised from the section with a focused UV laser. Sections of thickness 15 or 20 μm proved to be the most appropriate for the LM procedure.

Capture of the target was initially attempted by catapulting from a section mounted on a standard microscope glass slide into the cap of the collection vessel using laser-mediated tissue ablation (multiple laser pulses). However this method proved unsatisfactory as parts of the target remained attached to the surface of the slide, which compromised the estimation of the volume of the sample. Subsequently, laser pressure catapulting was used to effect the transfer from tissue mounted on membrane-covered slides. Here, a defocused laser pulse was used to catapult the sample into collection vessel cap. This method was only applicable for small target tissues, since the movement of bigger pieces was prevented by gravity. Finally, a micro-needle was employed to pick up and transfer the target, a method which also enabled the use of different collection vessels (plastic/glass tubes in different sizes) adjusted to the downstream biochemical analyses.

Various alternative materials were compared to act as the membrane covering the slide. The best results were obtained using PET (data not shown). Figure 1 illustrates the outcome of mounting 20 μm sections of seeds harvested 28 days after flowering on slides covered with a PET membrane. LM, in combination with the use of a micro-needle, allowed the isolation of the hypocotyl, and both the inner and the outer cotyledon tissue. Subsequent procedures for the extraction and the analysis of sugars, starch and lipid were adapted to suit a sample volume of ~0.008 mm^3^. An approx. five fold greater volume of tissue was required for the reproducible analysis of free amino acids, sugar phosphates, nucleotides as well as intermediates of glycolysis and citric acid cycle. For each measurement of metabolites and storage products, five technical replicates were taken from adjacent cross-sections of a single seed. Extractions and analyses of blank samples and samples containing only PET membrane were performed to provide an estimate of the background levels of analyte.

### Analysis of lipid content and fatty acid composition in LM samples

**Figure 2:**
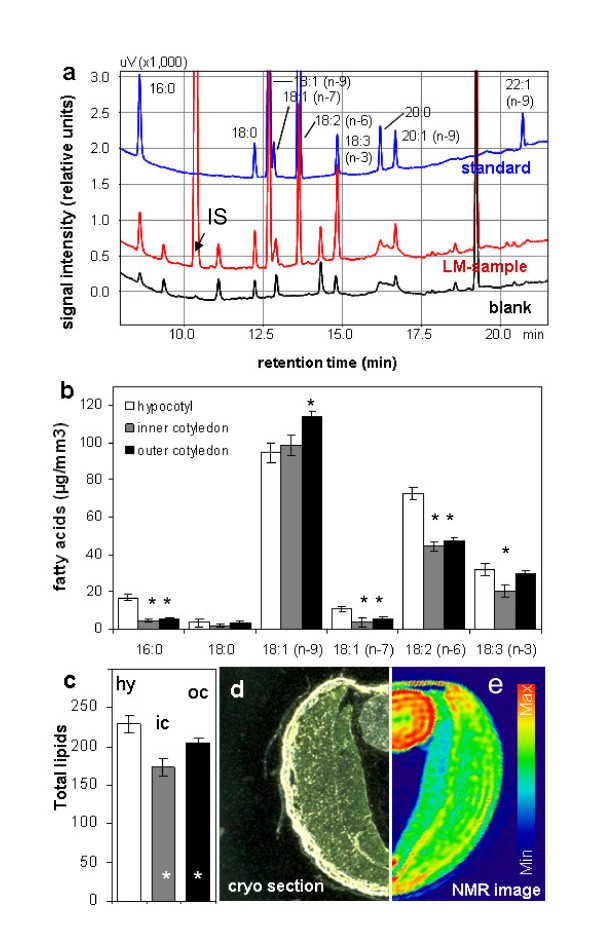
**Total lipids in laser-microdissected tissues of *B. napus *measured by gas chromatography**. (**a**) Typical chromatograms of lipid standard, LM-sample and blank; IS = internal standard added to tissue samples during extraction. (**b**) Fatty acids in LM-samples of hypocotyl, inner cotyledon and outer cotyledon of one dissected element per tissue type (minimum tissue amount ~0.0084 mm^3^). (**c**) Comparison of total lipids in hypocotyl, inner- and outer cotyledon tissue. (**d **&**e**) Tissue localisation within a cryosection and the adequate lipid distribution map (based on spin-echo NMR). Lipid levels are color-coded. Mean values +/- standard deviation are shown (n = 5). Stars in b and c indicate statistically significant differences versus hypocotyl according to t-test (p < 0.01).

Lipid extraction from LM samples was based on modifications of standard protocols (see Method section). Following extraction with organic solvents and transmethylation, the total lipid content in LM samples was assessed in the form of fatty acid methyl esters using gas chromatography (GC) (Figure 2). Where very small tissue samples are analysed, interference from contaminating impurities is a real issue [32]. As a precaution therefore, all plasticware (tips, tubes, etc.) was substituted with glass equivalents, pre-rinsed twice with solvent. This measure permitted the unequivocal detection of oleic and linoleic acids, and minimized the presence of impurities in the recovered fractions containing palmitic, stearic, vaccenic and linolenic acids (Figure 2a). A stable and low level of impurity was uniformly recorded from blank samples, and this background level was therefore subtracted from each sample reading, thereby avoiding the overestimation of individual lipid contents. The low standard deviations among chromatograph peak areas for each of the individual lipid species confirmed the experimental robustness of the analytical method (Figure 2b). A higher concentration of palmitic, vaccenic and linoleic acids was present in the hypocotyl compared with the cotyledons, while that of oleic acid was highest in the outer cotyledon and lowest in the hypocotyl. The least linolenic acid was present in the inner cotyledon, with similar concentrations present in the hypocotyl and outer cotyledon. When expressed in the form of total lipid, the three tissues differed markedly from one another (Figure 2c), with the hypocotyl being the most, and the inner cotyledon the least lipid rich. Application of NMR [for details see [33,34]] on intact seed of *Brassica napus *further confirms our LM-based results. The NMR-based lipid map based on frequency-selective radiofrequency pulses (spin echo) in Figure 2d demonstrates max lipid levels in hypocotyl (red colored) and min levels in inner cotyledon.

### Combined extraction and measurement of starch and soluble metabolites

To enable the simultaneous quantification of particular metabolites and starch, the sample was first collected using Vivaclear mini clarifying filter (0.8 μm PES membrane). Soluble metabolites were extracted with methanol, and then starch was recovered from the pellet remaining on the membrane.

#### Starch

**Figure 3:**
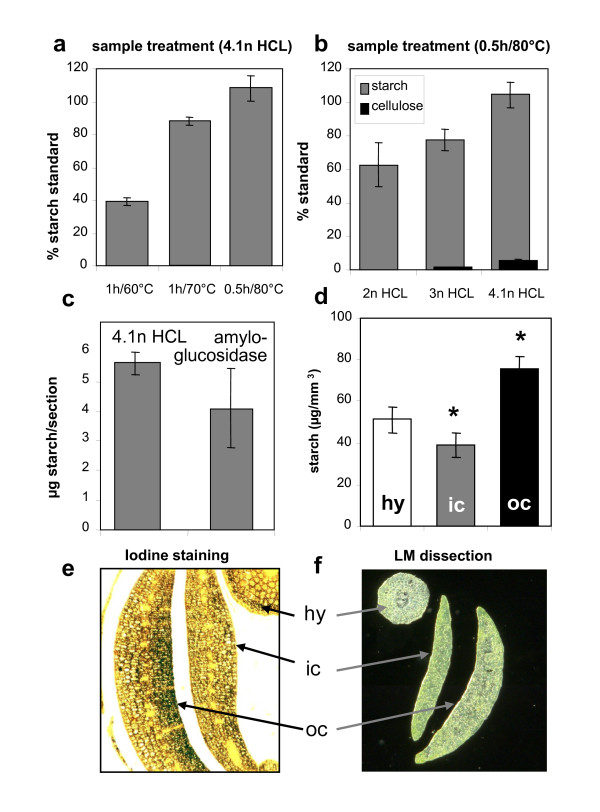
**Total starch in laser-microdissected tissues of *B. napus *as measured by ion chromatography**. (**a **&**b**) Effect of different HCl treatments on hydrolysis of starch and cellulose standards. (**c**) Level of starch detected in seed cross sections following either amyloglucosidase or HCl-mediated hydrolysis of starch. (**d**) Starch detected in dissected samples of 1 element per tissue type (minimum tissue amount ~ 0.0063 mm^3^). (**e**) Starch staining on a fixed seed cross section. Dark spots indicate accumulation of starch. (**f**) Laser dissected tissues showing hypocotyl (hy), inner cotyledon (ic) and outer cotyledon (oc). Mean values +/- standard deviation are shown (n = 5) in a-d. Stars in d indicate statistically significant differences versus hypocotyl according to t-test (p < 0.01).

The rapid and effective extraction procedure developed was based on treatment of the pellet remaining on the membrane in 4.1 M HCl for 0.5 h at 80°C. The resulting glucose was quantified using ion chromatography (IC) coupled to amperometric detection. The treatment completely hydrolysed starch (Figure 3a) but left cellulose largely unaffected (Figure 3b). Acid solubilization and amyloglucosidase-based hydrolysis produced comparable estimates for the quantity of starch present in the sample (Figure 3c), but the former method proved the more reproducible, since the resulting standard deviation was lower. A small degree of contamination (~0.4 ng glucose per μl extraction solution) was associated with the use of the Vivaclear filter, but this can be simply corrected by subtraction when estimating the starch level of an LM sample. The treatment of a PET-membrane-blank sample with HCl produced no signals that interfered with the subsequent starch measurement. Statistically higher levels of starch were present in the outer cotyledon (75.6 ± 6.3 μg/mm^3^) than in either the inner cotyledon (39.1 ± 5.8 μg/mm^3^) or the hypocotyl (51.2 ± 6.3 μg/mm^3^) (Figure 3d). These levels agree with those derived from histological staining for starch in a tissue slice of oilseed rape seed sampled at the same physiological stage as used in the LM analysis (Figure 3e). A preferential accumulation of starch in the outer cotyledon has also been noted in the *Vicia faba *seed [35]. These inter-tissue differences in starch accumulation could be transitory, as the developmental stage analysed here (28 days after flowering) coincides closely with the period of peak transient starch storage in the oilseed rape seed [28]. Based on the starch concentrations measured here using the LM technique, the mean starch content of the embryo at this stage was ~60 mg/g fresh weight.

#### Metabolites of core primary metabolism

**Figure 4:**
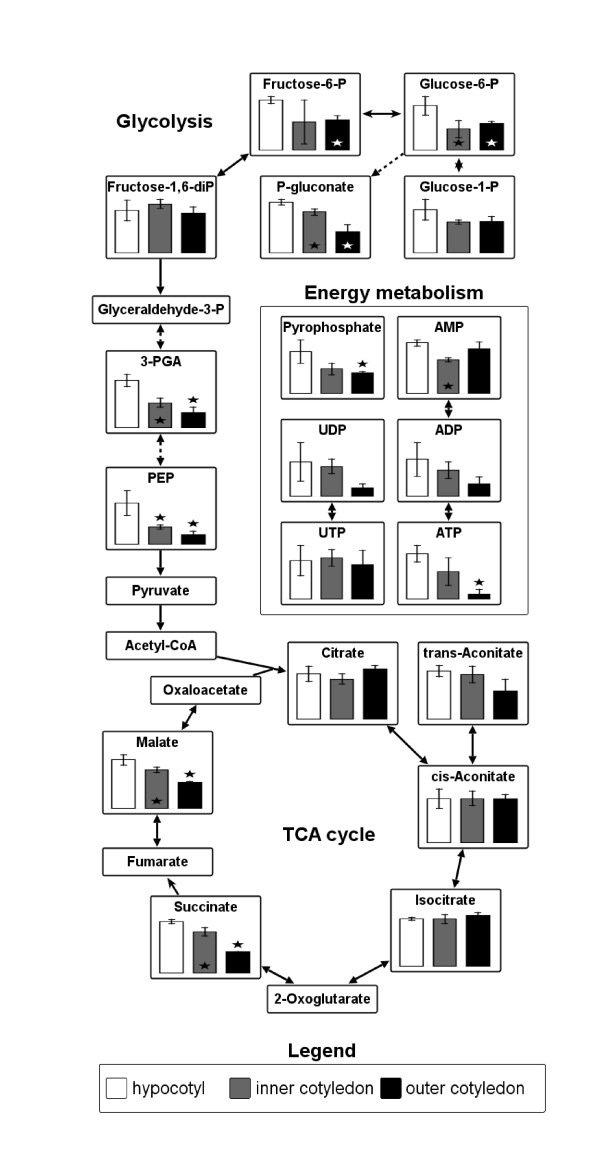
**Metabolic intermediates in laser-microdissected tissues of *B. napus *measured by LC/MS**. Levels of metabolites related to sugar breakdown (hexose-phosphates), glycolysis, citric acid (TCA) cycle and energy metabolism (nucleotides and pyrophosphate) were measured in LM-tissue of embryo (minimum tissue amount ~ 0.0063 mm^3^). ^13^C6-succinate was added prior extraction as internal standard. Data represent relative values and are given as mean +/- standard deviation (n = 3). Stars indicate statistically significant differences versus hypocotyl according to t-test (p < 0.05).

Metabolite profiling typically is based on mass spectrometry (MS) coupled to either GC or liquid chromatography (LC) [36]. While a number of GC/MS-based profiling techniques have been developed for use with LM tissues [24,36], they suffer from the problem that several compounds (e.g. most phosphorylated sugars) are either unstable or non-volatile, and thus are inaccessible. Thus we preferred to explore the potential of LC/MS-based metabolite profiling. The LC/MS protocol developed was shown to be sensitive enough to reliably detect the major compounds produced during primary metabolic pathways, specifically sugar breakdown, glycolysis, and the citric acid cycle, as well as nucleotides related to energy metabolism (Figure 4). The reduction of sugar phosphates and glycolytic intermediates occurred at a statistically different rate in the cotyledons compared to the hypocotyl. Although not pursued here, these metabolites could be readily quantified using external calibration procedures.

#### Soluble carbohydrates

**Figure 5:**
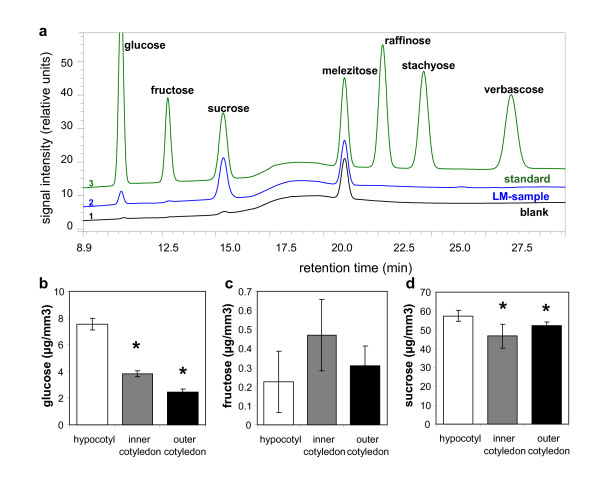
**Soluble sugars in laser-microdissected tissues of *B. napus *measured by ion chromatography**. (**a**) Typical chromatograms of sugar standard, LM-sample and blank. Melezitose was added during extraction to all samples as an internal standard. (**b-d**) Levels of glucose (b), fructose (c) and sucrose (d) in LM-samples of 1 element per hypocotyl, inner cotyledon and outer cotyledon (minimum tissue amount ~0.0063 mm^3^). Mean values +/- standard deviation are shown (n = 5). Stars in b-d indicate statistically significant differences versus hypocotyl according to t-test (p < 0.01).

Soluble carbohydrates were quantified by IC coupled to amperometric detection. Typical chromatograms derived from of an LM, a standard and a blank sample are presented in Figure 5a. Only traces of carbohydrates (60 pg/μl glucose and 0.65 ng/μl sucrose) were detected in the blank sample, and are assumed to have leached from the Vivaclear filter. The estimated carbohydrate concentrations in the LM samples were adjusted accordingly. At the 28 days after flowering stage, sucrose was the predominant soluble carbohydrate in the embryo, and was evenly distributed between the hypocotyl and cotyledons (Figure 5d). In contrast, there was a pronounced gradient of glucose concentration between the hypocotyl and the outer cotyledon (Figure 5b). Traces of fructose (Figure 5C) and some higher oligosaccharides (raffinose, stachyose and verbascose; data not shown) were also detected, as has also been noted in the *A. thaliana *embryo, where an increasing synthesis of higher oligosaccharides occurs as maturation proceeds [37].

#### Free amino acids

**Figure 6:**
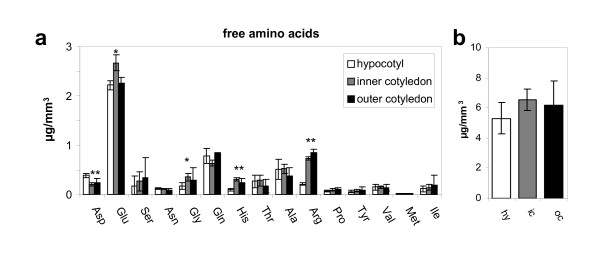
**Free amino acids in laser-microdissected tissues of *B. napus *measured by HPLC**. (**a**) Comparison of single free amino acids per analysed sample of 5 elements per tissue (minimum tissue amount ~0.036 mm^3^). (**b**) Total amount of free amino acids calculated as the sum over all detected amino acids. Mean values +/- standard deviation are shown (n = 5). Stars indicate statistically significant differences versus hypocotyl according to t-test (p < 0.01). Abbreviations: hy-hypocotyl; in-inner cotyledon, oc-outer cotyledon

HPLC was used to quantify the presence of 15 free amino acids in the LM samples. As most of these were present in very small concentrations, subtraction of blank sample levels was particularly important, and a somewhat larger size of LM sample was required. Relatively minor differences in amino acid composition were noted between the tissues (Figure 6a). Thus, glutamic acid was more abundant in the inner cotyledon, whereas arginine was least abundant in the hypocotyl. The most abundant amino acid across the whole embryo was glutamic acid. The free amino acid concentration in the hypocotyl was 5.3 ± 1.0 μg/mm^3^, compared to 6.6 ± 0.7 μg/mm^3 ^in the inner cotyledon (Figure 6b).

### Procedure for RNA extraction and amplification from laser microdissected tissues

To maintain comparability of transcript and metabolite analyses, our goal was to find an efficient, robust and technically simple method to extract RNA from cryosectioned seed-material, prepared in the same way as for metabolite measurement.

**Figure 7:**
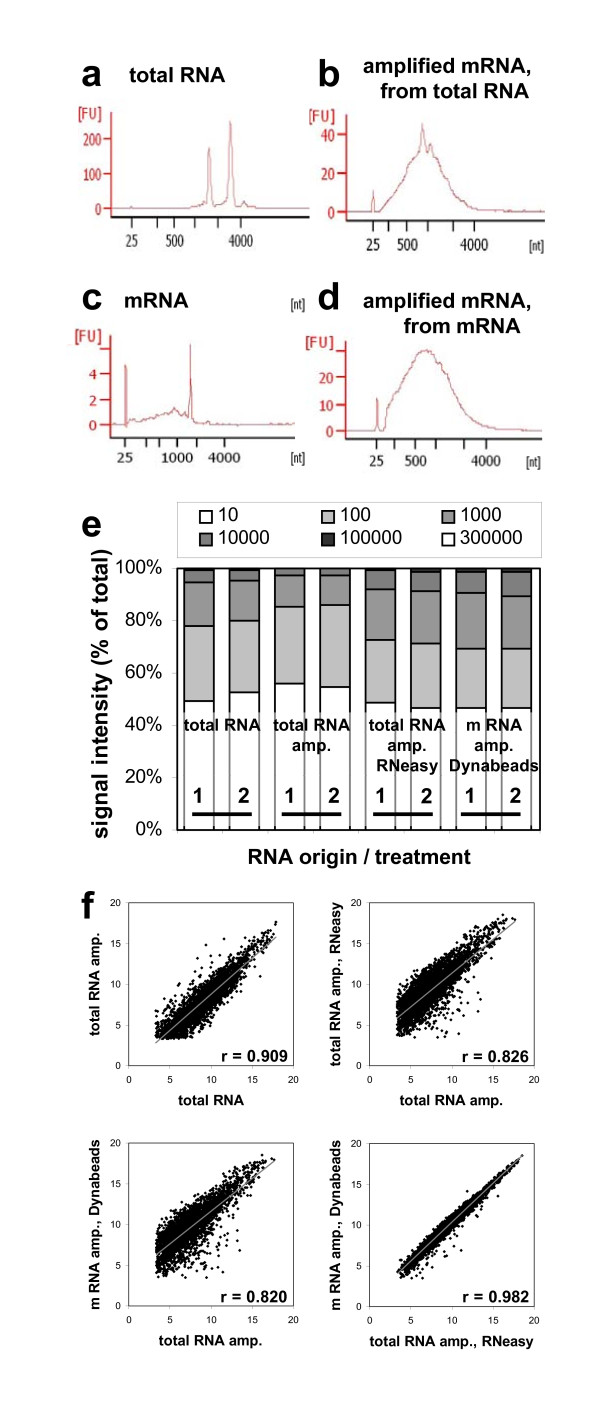
**Transcript analysis**. Closeness of expression signals obtained after microarray hybridisation of conventionally extracted total RNA and amplified RNA samples of conventional, column-based and bead-based extraction methods. (**a **&**c**) Electropherograms reflecting quality of total RNA, isolated with RNeasy^® ^(a) and mRNA, isolated with Dynabeads^® ^(c) from a 20 μm seed cryosection (43. DAF). (**b **&**d**) Electropherograms of mRNA from the same samples (a/c) after 2 rounds of amplification. (**e**) Portions of signal intensities obtained from each hybridisation and divided into six groups. Y-axis represent % of observation. (**f**) Scatterplots comparing log_2_-transformed means of expression signals from the different sample treatments. Means are filtered against low signal intensities (≤ 10) and high coefficients of variation (≥ 20%).

We tested different RNA extraction methods from whole 20 μm thick cross-sections: a column based total RNA extraction in comparison to a bead based mRNA extraction method. Both extraction methods resulted in sufficient amounts (131.9 ng total RNA, 2.7 ng mRNA) of high quality RNA (Figure 7 a/c) for at least two technical replicates for subsequent linear RNA amplification using Eberwine Method [38].

After two rounds of amplification we were able to increase the RNA amount from approximately 44 ng input total RNA up to 5.1/10.4 μg and accordingly from 1.1 ng input mRNA to 2.8/10.4 μg high quality mRNA (Figure 7 b/d). Based on the Agilent Bioanalyzer electropherograms, the obtained transcript lengths were estimated between 50 and 4000 nt with most abundant transcripts being about 1000 nt long.

Different methods of RNA isolation and amplification will introduce some degree of bias into the population of amplified RNA. For evaluation, we compared: (1) total RNA isolated from fresh frozen rapeseed using a conventional phenol/chloroform method (previously described in Heim et al. [39]) (T-NA); (2) the same RNA but diluted and amplified (T-AM); (3) poly (A) mRNA isolated from one microdissected rapeseed tissue slice (20 μm) using Dynabeads^®^, amplified (mD-AM); (4) total RNA isolated from one microdissected rapeseed tissue slice (20 μm) using RNeasy^®^, amplified (TQ-AM).

For technical repeat the isolated RNA was divided into two parts, which were then separately amplified for two rounds. Hybridisations of the samples were performed on a 4 × 44 K array representing 43.803 *Brassica *probes. Normalised absolute signal intensities, which are corresponding to the expression level of the probes represented by the array, were arranged as shown in Figure 7e. About 50% of signals showed background intensity (less than 10) in all experiments what could be caused by the temporary and tissue specific expression of some genes. Portion of signal intensities above 1000, from the samples produced by amplified RNA after conventional phenol/chloroform RNA isolation method (T-NA and T-AM), was smaller than those of the kit based methods (mD-AM and TQ-AM). This indicates that kit based methods are in general more suitable for the sensitive amplification procedure and transcriptome analyses. The comparison of signal intensities between unamplified RNA (T-NA) and diluted and amplified (T-AM) demonstrates a loss of some transcripts during amplification. Consequently microarray data resulting from different pre-processing's regarding amplification should not be compared within one analysis. But nevertheless, the kit based RNA extraction derived from microdissected tissues, together with amplification of RNA, gives more high-signal-intensity spots than conventional methods of RNA isolation.

We evaluated the reproducibility of this method and the consistency of the propagation of the amplification bias by comparing the expression profiles of hybridizations of two independently amplified RNA samples (Additional File 1). The high degree of correlation between independent replicates (r ~0.98), suggests that the amplification is highly reproducible and that the amplification bias is introduced consistently when the protocol is repeated.

Log_2_(ratio) plots of fluorescence intensity in non-amplified total RNA (T-NA) sample was compared with amplified (T-AM) and amplified targets were compared to each other: T-AM vs. TQ-AM, T-AM vs. mD-AM, TQ-AM vs. mD-AM for correlation analyses (Figure 7f). The scatter plots showed that the degree of signal variation was a function of the signal intensity, with the variation increasing as the signal intensities decreased. Correlation of expression intensities between amplified and unamplified transcripts, originated from the same sample, was high (*r *= 0.909). The small bias is probably caused by the loss of transcripts during amplification as mentioned above. The intermediate correlation in comparisons T-AM vs. TQ-AM, *r *= 0.826 and T-AM vs. mD-AM, *r *= 0.820 show again that the kit based RNA is not well comparable to the RNA of conventional extraction method. However the direct comparison of the two kit based extraction methods (TQ-AM vs. mD-AM) show a strong correlation (*r *= 0.982) although RNA is originated from two distinct tissue slices. As a consequence of this, both methods are well suited for further application on *B. napus *seeds.

Taken together, the both methods of RNA isolation from cryosectioned seed material gain sufficient high-quality RNA. Amplification procedure generates long transcript and yields greater than 100-fold RNA amplification. Microarray assays performed with amplified RNA demonstrate that the method results in low amplification bias, is highly reproducible and can be useful in conjunction with a variety of experimental systems.

## Conclusion

Standard extraction and analytical protocols have been modified to be applicable to LM tissues. As a result it has been possible to perform a parallel analysis of metabolic intermediates, major storage products and transcripts in LM tissues of seeds of *Brassica napus*. Protocols have been provided here for various analytical methods, differing in cost and analytical power. The approach was designed to enable a comprehensive analysis of LM tissues, and has been optimized for application to the important oilseed crop *Brassica napus*. We believe that the combination of metabolite profiling and the quantification of storage products, when combined with already established gene expression analyses from LM samples, should facilitate a comprehensive description of seed development under varying developmental/environmental conditions at a tissue-specific level, which would represent an important step in the context of both the systems biology and the biotechnology of oilseeds.

## Materials and methods

### Plant material

Plants of *Brassica napus *were grown on soil in a climate chamber at 16 h light/19°C and 8 h dark/16°C, 70% air humidity. Flowers were tagged at the time of opening for determination of the days after flowering (DAF).

### Cryosectioning

Seeds were frozen in liquid nitrogen and transferred to a cryotome (Bright Instrument Co Ltd, Huntingdon, England) cooled down to -20°C. By the means of Tissue-Tek^® ^O.C.T.™ Compount embedding medium (Sakura Finetek Europe B.V., Zoeterwoude, The Netherlands) the frozen seeds were glued onto the sample plate and cut into 15, 20 or 30 μm thick cross sections. Immediately, cryosections were mounted on plain superfrost microscope slides (Carl Roth KG, Karlsruhe, Germany) or different types of membrane-slides like 1.0 mm PET-membrane frame-slides, 1.0 mm PET-membrane glass slides or 1.0 mm PEN-membrane glass slides (Carl Zeiss Microimaging GmbH, Bernried, Germany) and stored until complete dryness for 7-10 days in the cryostat chamber at -20°C.

### Tissue preparation for metabolic analysis

Prior to laser microdissection, the freeze-dried cross sections were allowed to equilibrate to room temperature inside an airtight container to avoid condensation of moisture on the tissue. For microdissection, the target tissues were selected by the use of PALM^® ^RoboSoftware and dissected by the PALM^® ^MicroBeam System (Carl Zeiss Microimaging GmbH, Bernried, Germany). Following microdissection the tissue elements were picked by a microneedle and transferred into an 8 ml glass tube (Pyrex^®^, England) for fatty acid extractions. For the combined extraction of starch and soluble metabolites the dissected tissue elements were transferred directly on the 0.8 μm PES-membrane of a Vivaclear mini clarifying filter (Sartorius Stedim Biotech GmbH, Göttingen, Germany).

### Analysis of total lipids

The overall fatty acid composition of microdissected tissues was extracted and transmethylated according to the method of Miquel and Browse [40] with some modifications. As internal standard we used glyceryl triheptadecanoate and accordingly for GC heptadecanoic acid (Sigma-Aldrich Chemie GmbH, Steinheim, Germany). Samples were treated for 2 min with ultrasonic and then heated for 60 min at 80°C in 1 ml of 2.5% (v/v) H_2_SO_4 _and 2% (v/v) dimethoxipropane in methanol. After adding 200 μl saturated NaCL solution and 2 ml n-hexane, fatty acid methylesters (FAMEs) were transferred to the organic phase by intensive vortexing followed by centrifugation for 10 min at 1500 rpm. Subsequently the organic n-hexane-phase was transferred into a new 8 ml glass tube and extraction of the lower aqueous layer was repeated once. Afterwards 2 ml distilled water were added to the organic phase, vortexed, centrifuged (10 min/1500 rpm) and the organic layer was separated in a new glass tube where it was evaporated under nitrogen gassing and finally resuspended in 100 μl acetonitrile. For the whole extraction procedure it was necessary to avoid the use of plastics which were replaced by glass equivalents and additionally rinsed twice with distilled water and ethanol.

Analysis of FAMEs was carried out using a gas chromatograph GC-2014 equipped with auto injector AOC-20i and flame ionization detector (Shimadzu Corporation, Kyoto, Japan). The separation was performed on a 30 m × 250 × 0.25 μm DB-23 capillary column (Agilent Technologies, USA). The column temperature was set initially at 150°C (held for 1 min) then increased at 3°/min to 215°C, followed by 15°/min to 250°C which was held for 6 min. Identification and quantification of the detected fatty acids was done by comparison of retention times with FAME standards of different concentrations which were separated under same conditions. Signal intensities of impurities were subtracted by means of blank measurements. The amount of total lipids was calculated as the sum over all detected FAMEs.

### Combined extraction of soluble metabolites and starch

For the combined isolation of soluble metabolites and starch 200 μl 10% methanol were added to the dissected tissue elements on the 0.8 μm PES-membrane. Additionally we added 2 nmol melezitose as internal standard. After 2 min ultrasonic treatment, samples were cooled for 5 min on ice and centrifuged at 13000 rpm for 1 min. This extraction was repeated twice followed by concentration of the flow through under vacuum. Dry samples were redissolved in 100 μl 10% methanol and stored at -80°C. For extraction of starch, 100 μl 15% HCl were added to the tissue residue on the filter membranes and incubated 30 min at 80°C. After cooling, samples were centrifuged for 1 min at 13000 rpm and diluted with 60 μl 10% methanol.

### Analysis of soluble sugars and starch

Soluble sugars as well as glucose of hydrolysed starch were analysed using the Dionex ICS 3000 system coupled to an amperometric detector (Dionex, Idstein, Germany). Separation was performed on CarboPac™PA1 column (4 × 250 mm; Dionex, Idstein, Germany) under total flow of 1.3 ml/min distilled water (A) and 150 mM NaOH (B) in the following gradients: t = 0 min (91% A, 9% B); t = 15 min (60% A, 40% B); t = 18 min (100% B); t = 32 min (100% B); t = 32.5 min (91% A, 9% B); t = 38 min (91% A, 9% B) and column temperature of 30°C. Melezitose was used as internal standard and signal intensities of impurities were subtracted as means of blank measurements. Injection volume was set to 10 μl. Taken into consideration the tissue density (~1 g/ml) and volume-weighed contribution of the various tissues(hypocotyl, inner and outer cotyledons), the mean starch content of the embryo can be calculated and related to mean fresh weight.

### Analysis of free amino acids

Derivatisation of the samples was performed using the AccQ-Fluor™ Reagent Kit (Waters, USA) according to the manufacturer's instruction. Separation was accomplished by AccQ-Tag™ column (3.9 × 150 mm, Waters, Ireland) in a Summit HPLC-system (Dionex, Idstein, Germany) equipped with a fluorescence detector. For separation the column temperature was set to 37°C with a total flow of 1 ml/min. As eluents we used buffer (A) with 7 mM triethanolamine hydrochloride and 140 mM sodium acetate, (B) acetonitrile and (C) distilled water, in the following gradients: t = 0 min (100% A); t = 0.5 min (99% A, 1% B); t = 27 min (95% A, 5% B); t = 28.5 min (91% A, 9% B); t = 44.5 min (82% A, 18% B); t = 47.5 min (60% B, 40% C); t = 50.5 min (100% A) and t = 60 min (100% A). Excitation wavelength was 250 nm and emission wavelength 395 nm. Identification and quantification of the detected free amino acids was done by external calibration with an amino acid standard mix (Sigma-Aldrich Chemie GmbH, Steinheim, Germany) completed with asparagine and glutamine. Signal intensities of impurities were subtracted as means of blank measurements. The amount of total free amino acids was calculated as the sum of all detected amino acids.

### LC/MS-based metabolite profiling

Metabolic intermediates were analysed using the ICS 3000 system (Dionex, Idstein, Germany) coupled to a API 4000 triple quadrupole mass spectrometer (ABSciex, Darmstadt, Germany). Separation was performed on a IonSwift MAX-100 column (1 × 250 mm, Dionex, Idstein, Germany) under constant column temperature of 40°C and total flow of 150 μl/min. With sodium hydroxide as the eluent we used the following gradients: t = 0 min (5 mM); t = 10 min (5 mM); t = 16 min (12 mM); t = 28 min (25 mM); t = 32 min (100 mM); t = 38 min (100 mM); t = 42 min (5 mM) and t = 56 min (5 mM). The identification of the detected metabolites was done by specific MS/MS transitions described in detail elsewhere [41].

### Transcript analysis

Total RNA was extracted from whole seeds of 43 DAF by the conventional phenol/chloroform method as previously described [39]. Finally RNA was treated with RNase-free DNase I using TURBO DNA-free™ kit (Ambion, Austin, Texas). For mRNA extraction from a 20 μm thick cross section of 43 day old seeds Dynabeads^® ^mRNA DIRECT™ Micro Kit (Invitrogen, Karlsruhe, Germany) was used according to the manufacturer's protocol. Total RNA from a cross section was isolated using RNeasy^® ^Micro Kit (Qiagen, Hilden, Germany) with some modifications. 350 μl lysis buffer RLC was added to the cross section and incubated for 2 min at 56°C. Subsequently the lysate was transferred to a QIAshredder spin column (Qiagen, Hilden, Germany) and centrifuged for 2 min. The supernatant of the flow-through was mixed with 0.5 volume 100% ethanol and RNA extraction combined with on column DNA digestion was continued with step 6 according to the manufacturer's protocol pp39.

To get sufficient RNA for microarray analyses we performed a two-round linear amplification with Dynabeads^® ^mRNA, RNeasy^® ^total RNA and a diluted aliquot of conventional extracted total RNA, using C&E Version ExpressArt mRNA amplification Nano kit (Amptec GmbH, Hamburg, Germany) according to the manufacturer's protocol.

Quality and quantity of both the extracted and the amplified RNA was checked with Agilent 2100 Bioanalyzer, RNA 6000 Pico Kit and accordingly RNA 6000 Nano Kit (Agilent Technologies, Waldbronn, Germany) and in addition with NanoDrop™ 1000 (PeqLab GmbH, Erlangen, Germany).

Hybridisation of the 4 × 44 K microarray representing 43.803 *Brassica *probes (Agilent Technologies, Waldbronn, Germany) was performed by ATLAS Biolabs GmbH (Berlin, Germany). Normalised data were generated using the Agilents Feature Extraction Software (Version 10.5.1.1). For correlation analyses the normalised signal values were filtered against low signal intensities (≤ 10) and high coefficients of variation (≥ 20%) and subsequently transformed into Log_2 _values.

### Imaging techniques

Quantitative imaging of storage oils in rapeseed was performed using magnetic resonance imaging (17.6-T wide-bore superconducting magnet; Bruker BioSpin, Rheinstetten, Germany) equipped with actively shielded imaging gradients. The experiments were performed as described in detail in [33,34]. After non-invasive experiments seed were immediately frozen for further analysis. Starch staining was done using conventional staining with iodine solution as detailed in [42].

## List of abbreviations

DAF: Days after flowering; FAME: Fatty acid methyl ester; GC: Gas chromatography; HPLC: High-performance liquid chromatography; IC: Ion chromatography; LC/MS: Liquid chromatography-mass spectrometry; LM: Laser microdissection

## Competing interests

The authors declare that they have no competing interests.

## Authors' contributions

LB and HR conceived the study. SS, HT, LB, RR, and NH carried out the analysis. All authors contributed to draft the manuscript, and approved the final manuscript.

## Supplementary Material

Additional File 1 **Reproducibility of transcript analysis**. Scatter plot comparison of unfiltered signal intensities originated from technical replicates for the different sample treatments. Each axis of scatter plots represents independent amplification and hybridisation of one RNA sample. Correlation coefficient r reflects the high reproducibility of RNA amplification and array-analysis Click here for file
