# Supplementary figures and images for: A novel procedure for the quantitative analysis of metabolites, storage products and transcripts of laser microdissected seed tissues of Brassica napus
Source: Plant Methods. 2011 Jun 30;7:19. doi: 10.1186/1746-4811-7-19 (PMC3141804; doi:10.1186/1746-4811-7-19)

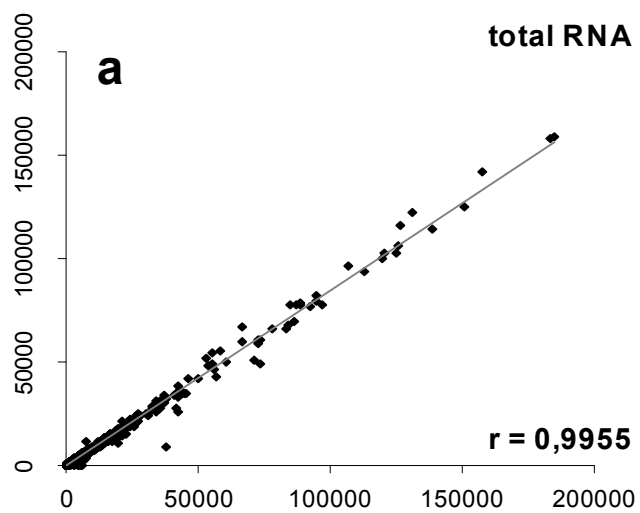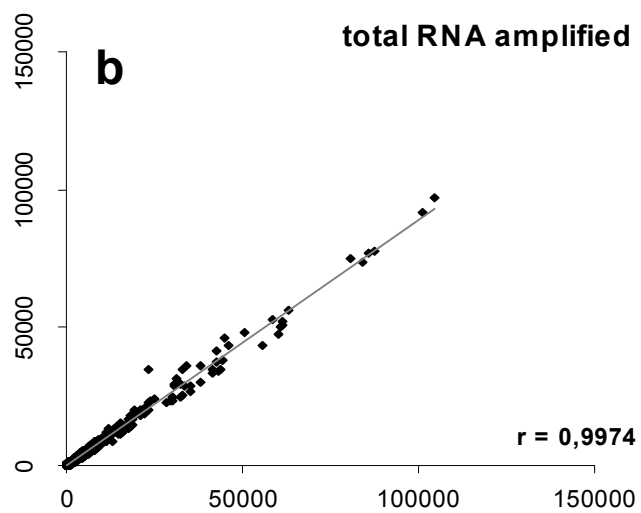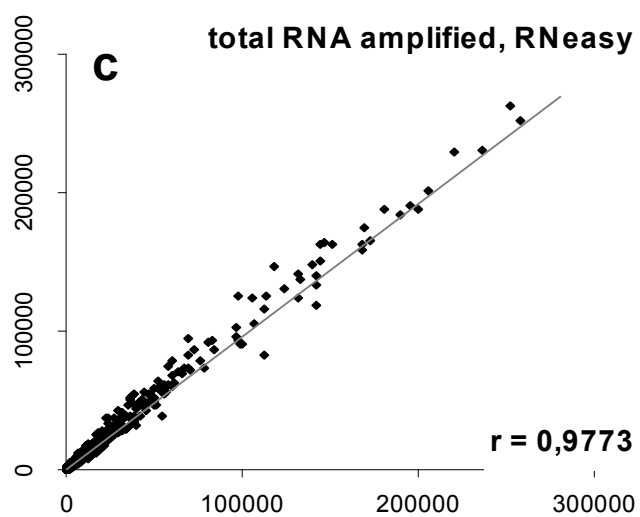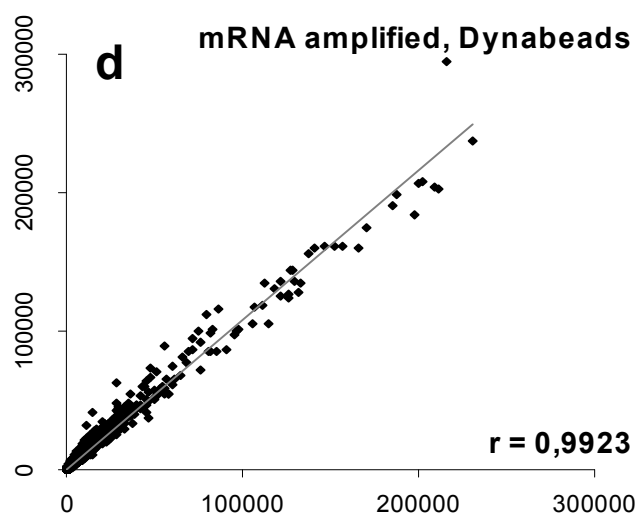

Supplement: Additional File 1 — Reproducibility of transcript analysis. Scatter plot comparison of unfiltered signal intensities originated from technical replicates for the different sample treatments. Each axis of scatter plots represents independent amplification and hybridisation of one RNA sample. Correlation coefficient r reflects the high reproducibility of RNA amplification and array-analysis [file 1746-4811-7-19-S1.PDF]
